# Editorial: New trends in esports and gaming: analyzing the impact of esports and video games on body composition, psychological state and health of gamers/players

**DOI:** 10.3389/fspor.2024.1514716

**Published:** 2024-11-18

**Authors:** Adrián Mateo-Orcajada, Mario Albaladejo-Saura, Frano Giakoni-Ramírez, Andrés Godoy, Raquel Vaquero-Cristóbal

**Affiliations:** ^1^ Facultad de Deporte, UCAM Universidad Católica de Murcia, Murcia, Spain; ^2^ Facultad de Educación y Ciencias Sociales, Instituto del Deporte y Bienestar, Universidad Andres Bello, Las Condes, Chile; ^3^ Grupo de Investigación en Educación Física, Salud y calidad de Vvida (EFISAL), Facultad de Educación, Universidad Autónoma de Chile, Temuco, Chile; ^4^ Research Group Movement Sciences and Sport (MS&SPORT), Department of Physical Activity and Sport Sciences, Faculty of Sport Sciences, University of Murcia, Murcia, Spain

**Keywords:** esports, videogames, health, psychology, body composition, physical activity

**Editorial on the Research Topic**
New trends in esports and gaming: analyzing the impact of esports and video games on body composition, psychological state and health of gamers/players

In this special issue, we have collected eight articles that provide insight into video games and esports from a healthy perspective for gamers. Esports have developed considerably and rapidly in many areas over the last decade. The majority of this development has been at the competitive level, with competition attracting global interest. However, there are other areas of interest within video games and esports, such as health, psychology, and body composition, due to the fact that players spend many hours per day sitting down, and the psychological demands are very high. This special issue brings together studies that have addressed the health-related, rather than the performance-related, side of this field.

More specifically, the first three articles focused on the psychophysiological changes and alterations that occur while playing video games in an amateur and professional environment. Thus, the first article showed how the mood of the players, despite not affecting the subsequent performance of amateur players, did change when they played successive games depending on the outcome of the previous game. This is important since they tend to play several video games on the same day. The second article described how 3–4 h of competitive video gaming can negatively affect the perceived physical exertion and perceived physical fitness of esports athletes, which could affect their health. A passive break may provide short-term regeneration, but it would not allow for full recovery. On the other hand, breaks that incorporate physical activity could mitigate additional negative consequences of sedentary behavior. This makes physical exercise and body awareness a crucial part of esports training. The third article highlighted the physiological stress responses of players during gaming. Gaming sessions lead to physiological changes such as increased HR, blood pressure, energy expenditure, and reduced HR variability. However, game genre, game outcome and fitness level had no effect on the stress response.

The fourth and fifth articles discussed the potential benefits and problems of playing video games and esports. The fourth article examined the usefulness of video games, specifically virtual reality games, as a tool to combat the sedentary lifestyle that commonly characterizes esports players. It was shown how virtual reality appears to alter the perception of exertion during physical activity, specifically reducing the perception of real exertion. The importance of this lies in the fact that perceived exertion is negatively related to physical activity adherence, so virtual reality could facilitate adherence to physical activity, with the health benefits that this would bring. The fifth article focused on one of the main problems with esports players, which is the perception of esports players themselves by their parents. Lack of parental support puts children at greater risk of missing out on the positive outcomes associated with esports, making them more prone to possible negative consequences. Parental support is determined by attitudes and perceived behavioral control. Negative attitudes revolve around concerns for their children's health and academic success, while a lack of perceived behavioral control is based on unfamiliarity with esports. Increased positive exposure to esports could contribute to more positive parental attitudes and improved esports competence.

The sixth and seventh articles addressed the importance of the problematic use of video games for the health of players, along with a very specific aspect that can contribute to this problematic use, such as tilt, which can also have negative consequences for psychological health. Tilt is a specific gaming term associated with frustration, rage, and deterioration of gaming ability. Tilt is a phenomenon in which players are triggered by a person or event in the game that generates frustration and other negative emotions that, in turn, start to negatively impact decision-making and overall gameplay. The sixth article presented a new methodology for analyzing indicators of gaming behavior, with the aim of improving the diagnosis and understanding of internet gaming disorder. To do so, behavioral telemetry data was used to extract emotional states, providing a nuanced understanding of player behavior and emotion regulation. This tool can assist healthcare professionals in the diagnosis and monitoring of the therapeutic process, helping to solve some of the problems associated with traditional methods of assessing internet gaming disorder. In addition, the metrics and visualizations can also inform therapists about the problematic behaviors and gaming habits of each gamer, allowing for personalized treatment tailored to the individual and their needs. In light of the fact that tilt is one of the major problems affecting the psychological state of players, the authors of the seventh article presented a specific questionnaire to measure this variable. This questionnaire allowed for the conceptualization and quantification of the tilt phenomenon, laying the groundwork for exploring its intricate relationships with other variables of interest. Thus, tilt was defined as a construct characterized by a state of frustration that escalates into anger, resulting in diminished performance, attention, and recurrent negative thoughts about errors. This study also introduced a valuable and promising tool for future research efforts on the psychological experiences of esports players, transcending diverse cultural contexts.

Finally, the eighth article discussed the perception of the use of performance enhancers in the esports context. The competitive gaming community generally distinguishes between potential performance enhancers and is more concerned with “hard” drugs, pharmaceuticals, and brain stimulation interventions. Socially acceptable drugs and foods or food supplements appear to be more accepted. This affects the perception of fairness, which is key to the competition being seen as legitimate. If an institution (e.g., a tournament organizer) can ensure a competition that is widely perceived as fair, both the organizer and the outcome are more likely to be perceived as legitimate.

